# In-hospital prognosis and long-term mortality of STEMI in a reperfusion network. “Head to head” analisys: invasive reperfusion vs optimal medical therapy

**DOI:** 10.1186/s12872-017-0574-6

**Published:** 2017-05-26

**Authors:** C . García-García, N. Ribas, L. L. Recasens, O. Meroño, I. Subirana, A. Fernández, A. Pérez, F. Miranda, H. Tizón-Marcos, J. Martí-Almor, J. Bruguera, R. Elosua

**Affiliations:** 1 Cardiology Department, Hospital del Mar, Parc de Salut Mar-IMIM, Barcelona, Spain; 2 Cardiology Department, Hospital Universitari Germans Trias i Pujol, Carretera Canyet s/n, 08916 Badalona, Spain; 3 CIBER de Enfermedades Cardiovasculares (CIBERCV), Barcelona, Spain; 4 Ph Program in Internal Medicine, Universitat Autònoma de Barcelona, Barcelona, Spain; 5 Heart Diseases Biomedical Research Group, IMIM (Hospital del Mar Medical Research Institute), Barcelona, Spain; 6 IMIM (Hospital del Mar Medical Research Institute), Cardiovascular Epidemiology and Genetics Group (EGEC), REGICOR Study Group, Barcelona, Spain

**Keywords:** Reperfusion network, AMI prognosis, Long-term mortality, Optimal medical therapy, Reperfusion therapy

## Abstract

**Background:**

ST Segment Elevation Acute myocardial infarction (STEMI) preferred treatment is culprit artery reperfusion with primary percutaneous coronary intervention (PPCI). We ought to analyze the benefit of early reperfusion vs. optimal medical therapy in STEMI before and after the set-up of a regional STEMI network that prioritizes PPCI.

**Methods:**

Between January 2002 and December 2013, 1268 STEMI patients were consecutively admitted in a University Hospital. Patients were classified in two groups: pre-STEMI Network (January 2002–June 2009; *n* = 670) and post-STEMI network (July 2009–December 2013; *n* = 598). Vital status was available at 2-year follow-up.

**Results:**

The STEMI network increased reperfusion (89.2% vs 64.4%, *p* < 0.001) mainly using PCI (99.0% vs 43.9%, *p* < 0.001). In univariate analysis, in-hospital mortality was significantly lower in the post-STEMI network period (2.51% vs. 7.16%, *p* < 0.001). After multivariate adjustment, including age, sex, comorbidities, severity and reperfusion therapy, a trend to a lower in-hospital mortality was observed (post-Network OR: 0.50, 95% CI:0.16–1.59, *p* = 0.24); this trend disappeared when optimal medical therapy was included in the model (post-Network OR: 1.14, 95% CI:0.32–4.08, *p* = 0.840). No differences in 2-year mortality were observed (post-Network HR: 0.83; CI 95%: 0.55–1.25, *p* = 0.37).

**Conclusion:**

A STEMI network with PPCI 24/7 improved reperfusion therapy, resulting in an increase on PPCI. Despite in-hospital mortality decreased with a STEMI network, 2-year mortality remained similar in both periods, pre- and post-Network. Optimal medical therapy could be as important as reperfusion therapy in a STEMI reperfusion network.

## Background

Primary percutaneous coronary intervention (PPCI) is the choice reperfusion therapy for ST-elevation acute myocardial infarction (STEMI) when performed at the right time [1, 2]. Reperfusion networks, which have been defined and established to optimize reperfusion therapy in STEMI patients [3–5], have achieved a reduction in reperfusion times and an increase in the proportion of patients receiving PPCI [6, 7]. However, the information about short- and long-term prognosis of patients included in a “real life” STEMI network and about the predictors of prognosis in these patients is scarce. The mortality of acute coronary syndromes has been reduced in the last years, and this decrease has been related not only to invasive or revascularization procedures but also to pharmacological treatments [8–11]. In stable coronary artery disease patients, optimal medical therapy (OMT) and angioplasty have similar beneficial effects [12–14], but the relative benefits of OMT in STEMI patients compared to those related to reperfusion therapy have not been well established. The aim of our study was: 1) to analyze the STEMI Reperfusion Network on in-hospital prognosis and 2-year mortality and 2) to compare the relative benefits of improving reperfusion therapy vs. optimal medical therapy in a consecutive population of STEMI patients in the last 11 years.

## Methods

### Study design

This is a prospective hospital register of STEMI patients with a long-term vital status follow up. All STEMI patients aged >18 admitted in the Coronary Care Unit of a University hospital from January 2002 to December 2013 were prospectively and consecutively included. The study was designed and implemented in accordance with Guidelines for Good Clinical Practice and with the ethical principles laid down in the Declaration of Helsinki. All participants gave their written consent to participate in the study. The study was approved by our institution Ethics Committee, the CEIC-Parc de Salut Mar with reference number 2012/4806/I.

### Variables of interest and STEMI management

Demographic variables and comorbidities such as history of hypertension, diabetes, hypercholesterolemia, smoking, and previous angina were prospectively collected. Clinical characteristics of the event were recorded, including ischemia times, AMI location and complications such as the development of pulmonary edema or cardiogenic shock or the presence of malignant arrhythmias. In addition, information about the management of the acute event, including medical treatments during the hospital stay, reperfusion therapy (including both thrombolysis or PPCI), type of reperfusion therapy (thrombolysis or PPCI) and invasive procedures (coronary angiography, mechanical ventilation), was also collected.

Patients’ care followed the current clinical practice guidelines for STEMI patients at the time of the study [15–17], but reperfusion therapy was applied according to the STEMI Code instruction [4] as a regional Reperfusion Network. In our study there was no standard care for patients. All treatments were performed under the physicians’ medical criteria depending on clinical patients’ situation.

STEMI Reperfusion Network was initiated in Catalonia in June 2009 with the purpose of reaching an optimal reperfusion therapy with PPCI [4]. Before the establishment of the STEMI Network (June 1st, 2009–pre-Network period), PPCI was performed in our hospital in STEMI patients only during working hours; thrombolytic therapy during on-duty time. After June 2009 (post-Network period), PPCI was the elective reperfusion therapy in STEMI patients. During working hours (8 am-20 pm), PPCI was performed in our hospital and patients first admitted in our hospital during on-duty time were transferred to another PPCI capable centre near our institution. Depending on the period of admission, patients were classified in two groups: pre-Network (January 2002 to May 2009) and post-Network (June 2009 to December 2013).

### Events of interest

Events of interest were defined as in-hospital and 2-year mortality. In order to identify long-term fatal cases, we accessed the National Death Registry. This is an exhaustive and mandatory official database which collects individual data of all the deceased in Spain from 1987 up to now. This database, promoted by the Spanish Health Ministry, provides public institutions (healthcare administrations, research centers) with information regarding vital status and date of death, although it does not indicate the specific cause of death. We linked our data with the National Death Registry. We assumed that study participants who did not appear in this registry were alive at the end of the follow-up.

### Statistical analysis

In the comparison of study groups (pre and post-Code), analysis of variance or Kruskall-Wallis test were used for continuous variables and the Chi-square test for categorical variables. Unconditional logistical regression and Cox regression were used to determine the association between comorbidities, reperfusion therapy and in-hospital or long-term mortality, with adjustment to the identified confusing variables. We tested the interaction between the use of reperfusion therapy, medical therapy and period of admission in order to evaluate in-hospital prognosis as well as after 2 years. *P* values lower than 0.05 were considered statistically significant.

In order to evaluate a mortality time trend along the period, day of admission was also incorporated in the Cox regression model as a spline term to accommodate a possible non-linear effect.

## Results

The study included 1268 consecutive STEMI patients. These patients were classified in two groups: pre-Code (*n* = 670) and post-Code (*n* = 598). The patients’ demographic and clinical characteristics are shown in Table 1. The proportion of smokers and peripheral artery disease was higher in the post-Network period whereas the proportion of diabetes mellitus was higher in the pre-Network period.

**Table 1:** Demographic and clinical characteristics of patients included in the study according to the two periods analyzed

	Pre-network *N* = 670	Post-network *N* = 598	*P* value
Age* (SD)	62.6 (13.6)	63.6 (13.1)	0.189
Men	76.1%	75.7%	0.929
Smoker	41.0%	48.0%	0.029
Hypertension	51.9%	57.3%	0.062
Dyslipidaemia	47.4%	50.7%	0.267
Diabetes mellitus	26.9%	23.9%	0.001
Peripheral vascular disease	4.9%	8.6%	0.013
Family history of CAD	11.2%	10.8%	0.789
Previous AMI	10.7%	11.2%	0.865
Killip III-IV at admission	10.3%	8.3%	0.075

*SD* standard deviation, *AMI* acute myocardial infarction, *CAD* coronary artery disease

### STEMI management

Reperfusion therapy increased in the post-Network period (89.2% vs. 64.4%). Among those treated with reperfusion there was an important increase in the use of PPCI (99% in the post-Network vs. 43.9% in the pre-Network period) with a subsequent decrease in the use of thrombolytics (1% vs. 56.1%). The changes in the reperfusion therapy strategy were associated with a slight increase in the ischemia time: median time from pain onset to reperfusion performance was 165 min (105–325 min) vs. 186 min (130–284 min) in pre- and post-Network periods respectively, *p* < 0.001. There were no changes in time from pain onset to monitoring: pre-Code 130 (60–258 min) vs. post-Network 90 (45–201 min), *p* = 0.254.

Medical therapy and procedures during hospital stay are shown in Table 2. There was an important increase in the use of evidence-based drugs such as statins, beta-blockers or angiotensin converting enzyme inhibitors. Above all, an increase in the use of dual antiplatelet therapy (clopidogrel and the new adenosine phosphate inhibitors), which could be associated to the extended use of PPCI in the post-Network period, was also observed. Although comprehensive data about length of dual antiplatelet therapy were not available for all patients, European Society of Cardiology STEMI guidelines recommendation about length of antiplatelet therapy (1) were systematic followed in all patients. Dual antiplatelet therapy (aspirin plus clopidogrel, ticagrelor or prasugrel) were prescribed during 1 year in all patients (either bare metal or drug eluting stents). After the first year, aspirin was the only antiplatelet therapy in treatment.

**Table 2:** Medical therapy and procedures used in the two periods

Drugs	Pre-network *N* = 670	Post-network *N* = 598	*P* value
Aspirin	96.6%	99.3%	0.001
Clopidogrel	54.3%	79.9%	<0.001
Ticagrelor	----	27.9%	----
Prasugrel	----	3.3%	----
GP IIb/IIIa Inhibitors	16.1%	13.1%	0.252
Heparin	82.1%	93.9%	<0.001
Betablockers	69.3%	81.2%	<0.001
Statins	89.7%	98.0%	<0.001
ACE inhibitors	70.1%	77.8%	0.003
Nitroglicerin	40.6%	37.2%	0.241
Eplerenone	-----	17.9%	-----
Mecanichal ventilation	8.5%	8.0%	0.870
Echocardiogram	40.3%	56.0%	<0.001
Coronary angiography	55.2%	99.5%	<0.001
IABP	4.8%	4.1%	0.680
*Swan-Ganz* cathether	7.8%	5.1%	0.080
Reperfusion	64.4%	89.2%	<0.001
PPCI	43.9%	99.0%	<0.001
Thrombolysis	56.1%	1.0%	<0.001

*ACE* angiotensin converting enzyme, *IABP* intra-aortic balloon pump, *PPCI* primary percutaneous coronary intervention

### In-hospital prognosis

In-hospital prognosis and complications are shown in Table 3. There was a reduction in complete atrioventricular block and a non significant trend to a lower Killip grade III-IV in the post-Nework period. An important decrease in in-hospital mortality (65%) was observed in the post-Network period (2.51% vs. 7.16%, *p* < 0.001).

**Table 3:** In-hospital complications and prognosis in the two periods analyzed

	Pre-network *N* = 670	Post-network *N* = 598	*P* value
Reinfarction	2.5%	1.5%	0.450
Ventricular fibrillation	4.5%	5.8%	0.327
Complete AV block	8.7%	5.5%	0.040
Flutter/Atrial fibrillation	6.3%	4.5%	0.211
Septal rupture	1.2%	0.7%	0.501
Papillar muscle rupture	0.7%	0.5%	0.729
Free wall rupture	0.7%	0.7%	1.000
Killip -Maximum, III-IV	13.7%	11.6%	0.349
In-hospital mortality	7.16%	2.51%	<0.001

*AV* atrio-ventricular

**Fig. 1:**
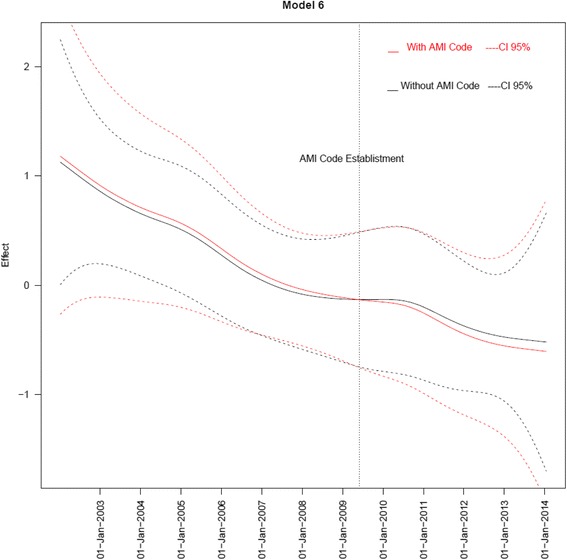
In-hospital mortality risk trend in the period 2003–2014. Multivariate Model 6 with and without including AMI Network as a binary variable

However, a significant decrease trend in in-hospital mortality was observed in the period analyzed (Fig. 1). Therefore, in order to analyze the effect of the reperfusion network on in-hospital mortality and the potential variables that could be involved in this effect, we included this mortality decreasing trend in the multivariate models. We observed a trend towards a decrease in in-hospital mortality in the post-Network period compared to the pre-Network period even when the model was adjusted by age, sex (Model 1), comorbidities (Model 2), severity (Model 3) and reperfusion (Model 4) with ORs ranged between 0.45 and 0.59 (Table 4). Noteworthy, when the model was further adjusted by optimal medical therapy (aspirin, statins, beta-blockers and angiotensin converting enzyme inhibitors), the benefit of the post-Network period disappeared totally (Table 4).

**Table 4:** Association between the STEMI-Code period and in-hospital mortality in different multivariate models

	Pre-network *N* = 670	Post-network *N* = 598	*P* value
		OR (CI 95%)	
Model 1	1	0.45 (0.15; 1.37)	0.160
Model 2	1	0.48 (0.16; 1.49)	0.207
Model 3	1	0.59 (0.18; 1.95)	0.385
Model 4a	1	0.57 (0.16; 1.97)	0.375
Model 4b	1	0.50 (0.16; 1.59)	0.239
Model 5	1	1.19 (0.30; 4.76)	0.805
Model 6	1	1.14 (0.32; 4.08)	0.840

Model 1: Adjusted by age and sex

Model 2: Model 1 plus hypertension, diabetes and smoke

Model 3: Model 2 plus Killip grade III-IV at admission

Model 4a: Model 3 plus reperfusion (including both thrombolysis or PPCI)

Model 4b: Model 2 plus reperfusion (including both thrombolysis or PPCI)

Model 5: Model 4a plus medical therapy (Aspirin, beta-blocker, ACE-inhibitors and statins)

Model 6: Model 2 plus medical therapy (Aspirin, beta-blocker, ACE-inhibitors and statins)

### Long-term mortality

**Fig. 2:**
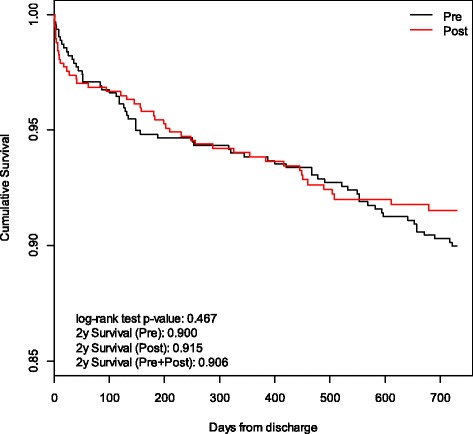
Kaplan-Maier 2-year cumulative survival curves in the STEMI pre-Network and post-Network periods

There was no difference in 2-year mortality among acute phase survivors between the two analyzed periods (10% pre-Network vs. 8.5% post-Network, *p* = 0.467). Kaplan Maier curves with cumulative 2-year mortality rates are shown in Fig. 2.

## Discussion

We analyzed the impact of the establishment of a reperfusion network, the STEMI Code, on the management and prognosis of STEMI patients in a prospective and consecutive hospital registry. In the post-Network period, reperfusion therapy was performed in almost 90% of STEMI patients, a significant increase compared to the pre-Network period, mainly due to an increase in the practice of PPCI. Furthermore, an important improvement in evidence-based medical treatment use (antiplatelet therapy, statins, beta-blockers or angiotensin converting enzyme inhibitors) was observed in the post-Network period. In-hospital mortality decreased after the establishment of the STEMI Network. This decrease seems to be mainly related to the optimization of medical treatment rather than to the increase of reperfusion. Two-year mortality was similar in both periods.

### Reperfusion therapy and ischemia times

In our series, the STEMI Network had an important impact on the reperfusion therapy rate and strategy. Reperfusion therapy was performed in nearly 90% of all STEMI patients, more than other national registries [18] and similar to the best European countries in STEMI acute phase reperfusion [19] like Czech Republic, a small country with a huge and experienced AMI network. Moreover, we also observed a change in the reperfusion strategy that was almost exclusively based on PPCI in the post-Network period, similar to what occurred in the Czech Republic registry [19].

Reperfusion therapy strategy is one of the most important factors to improve AMI prognosis, but time to reperfusion therapy is a main pillar too [20]. In our study we report an important increase in reperfusion rate and in the use of PPCI instead of thrombolysis, with a slight increase in time from pain onset to reperfusion performance (21 min). Our global ischemia time is similar to that reported in other European countries [21] or better than that reported in other recent studies [19]. Although some registries have confirmed the benefit of minimizing total ischemia time to improve in-hospital and long term prognosis [20], other studies have recently shown that short variations in ischemia time were not enough to modify in-hospital STEMI prognosis [22].

### In-hospital mortality

The establishment of the STEMI Network was associated with an in-hospital mortality decrease among the lowest compared to most developed European countries [23]. When we tried to identify the variables which caused that decrease, reperfusion therapy was not the main factor. The use of evidence-based medical therapy could be another potential explanation as has been reported in other recent STEMI registers [18]. The use of aspirin, beta-blockers, ACE inhibitors and statins in our series increased and could be considered at least as optimal as those prescribed in the Courage Trial [12], a study that proved a similar benefit of this optimal medical therapy compared to angioplasty in stable patients with angina. Both the introduction of this medical therapy in the multivariate model (model 5 and 6; Table 4) and the decrease of the benefit in the post-Code period imply that optical medical therapy could be one of the main variables related to the lower mortality of STEMI patients in the post-Code period. This fact suggests that optimal medical therapy could be as important as both the observed reperfusion therapy rate increase and the PPCI reperfusion strategy. These findings need careful validation in bigger observational studies.

### Long-term mortality

There were no differences in 2-year mortality rate among survivors to the STEMI acute phase between both periods, although our 2-year mortality is similar to the 1-year mortality rate reported in other recent studies [24]. Unfortunately, we had only information related to the vital status but not to the cause of death in fatal cases. No information about other prognosis variables, such as re-infarction or the need of revascularization was available. However, the main cause of death in the first year after an AMI is mainly related to cardiovascular events, as we can see in recent registries or important randomized studies [24, 25].

On the other hand, the benefits of PPCI vs thrombolytic therapy out of the acute phase could be more related to a lower re-infarction rate or to the need of revascularization than to prevent cardiovascular death. The reduction in long-term mortality could be due to optimal control of cardiovascular risk factors and the use of evidence-based medical therapy [26], especially in high risk patients [27].

## Study limitations

This is a single centre register that includes a limited number of patients, which limits the statistical power of the study and the capability to show statistically significant results. However, our results suggest a significant clinical association between the STEMI-Network and lower in-hospital mortality. Although the data come from a single centre and could limit the external validity of the results, the internal validity is guaranteed by the accuracy, homogeneity and consecutiveness of the data collection. Other study limitations are related to the long term follow-up; we had information concerning the discharge treatment but we lack information on long-term compliance to medical therapy and on the type of stent used during PPCI (bare metal or drug eluting stent) that could also be associated with the outcomes of interest of the study.

## Conclusions

The STEMI Code network increases reperfusion therapy rate and changes the reperfusion strategy that is mainly based on PPCI. In-hospital mortality of STEMI patients has decreased in the last 11 years probably due to the improvement in reperfusion therapy and evidence-based medical therapy optimization. Even in the setting of STEMI reperfusion networks, our results emphasize the relevance of optimal medical therapy. The establishment of STEMI Code network does not seem to be enough to reduce long-term mortality of STEMI patients.

## Abbreviations

OMT: Optimal medical therapy
PPCI: Primary percutaneous coronary intervention
STEMI: ST-elevation acute myocardial infarction
